# Surviving COVID-19: patients’ experiences of care and path to recovery

**DOI:** 10.1080/17482631.2024.2301953

**Published:** 2024-01-07

**Authors:** Rakel Eklund, Lisa Hjelmfors, Sophia Nyquist, Josefin Sveen, Michael Hultström, Miklos Lipcsey, Robert Frithiof, Ewa Wallin, Ing-Marie Larsson, Filip K. Arnberg, Lotti Orwelius

**Affiliations:** a National Centre for Disaster Psychiatry, Department of Medical Sciences, Uppsala University, Uppsala, Sweden; b Department of Women’s and Children’s Health, Uppsala University, Uppsala, Sweden; c Department of Health, Medicine and Caring Sciences, Division of Society and Health, Linköping University, Linköping, Sweden; d Department of Addiction Medicine, Uppsala University Hospital, Uppsala, Sweden; e Department of Surgical Sciences, Anaesthesiology and Intensive Care Medicine, Uppsala University, Uppsala, Sweden; f Centre for Crisis Psychology, University of Bergen, Bergen, Norway; g Department of Medical Cell Biology, Integrative Physiology, Uppsala University, Uppsala, Sweden; h Department of Surgical Sciences, Hedenstierna Laboratory, Uppsala University, Uppsala, Sweden; i Department of Intensive Care, Linköping University Hospital, Linköping, Sweden; j Biomedical and Clinical Sciences, Linköping University, Linköping, Sweden

**Keywords:** COVID-19, intensive care, psychosocial well-being, psychosocial support, recovery, rehabilitation

## Abstract

**Purpose:**

To examine patients’ experiences of receiving care on an ICU for COVID-19 and the subsequent rehabilitation process.

**Methods:**

An explorative and inductive design was used. Participants were recruited from two university hospitals in Sweden. Patients admitted to the ICU due to COVID-19 from March 2020 to April 2021, who enrolled in the ICU follow-up, and understood and spoke Swedish were invited to participate. In total, 20 participants completed a semi-structured interview, of whom 18 were included in the thematic analysis.

**Results:**

The analysis resulted in two themes: “An isolated world with silver linings” and “Recovery in the wake of the pandemic”. Findings show that patients cared for on an ICU for COVID-19 during the pandemic felt safe but experienced a sense of vulnerability. After discharge, physical rehabilitation was a slow process with frustrating day-to-day fluctuations. Mentally, participants felt isolated, fatigued, and emotionally sensitive. Patients reported that love and support from family and friends were crucial for the recovery process.

**Conclusions:**

This study highlights the challenges of recovering from COVID-19, emphasizing the importance of continued support from health care, public services, family and friends. It provides important insights into patients’ experiences and can inform future healthcare strategies and policies.

## Introduction

The COVID-19 pandemic has had a profound impact on individuals, communities and societies worldwide. While the focus was initially on the acute effects of the virus, such as hospitalization and death, greater attention is now being given to the lingering effects of COVID-19 that people experience (Parotto et al., 2021). Many patients recovering from the virus, particularly patients who were cared for in intensive care units (ICU), have reported a range of persistent physical, cognitive and emotional symptoms that have implications for their health-related quality of life and well-being. Despite ongoing efforts to address these issues, there is still a lot we do not know about the recovery process after a SARS-COV-2 infection, and there have been uncertainties about which the health care services that should be offered to patients to improve their recovery from severe COVID-19 (Goodwin et al., 2021; O’Brien et al., 2021).

The long-term consequences of COVID-19 are being increasingly recognized; for example, it has been recommended that therapeutic trials for hospitalized patients do not use health status at discharge as a primary end point (Douin et al., 2022). Many patients who survive COVID-19 continue to experience a range of physical, neurological and psychological symptoms for weeks, months or even longer after their initial infection, which can have a significant impact on their daily lives, particularly among those who required intensive care for severe infections (Global Burden of Disease Long COVID Collaborators et al., 2022; Larsson et al., 2023; Sevin & Ely, 2022). These symptoms have been referred to as *post-COVID* or *long-term COVID*, although it is important to recognize that any condition that requires intensive care can lead to negative consequences which significantly impact daily life and well-being (Brown et al., 2019; Hodgson et al., 2022). In some cases, individuals may be unable to return to work or engage in their usual activities due to persistent symptoms. In addition, the psychological impact of experiencing ongoing symptoms can be significant, causing distress and reduced overall health-related quality of life (Hellemons et al., 2022).

The causes of persistent poor psychological health after COVID-19 are not clearly understood (Townsend et al., 2021). Increased knowledge about the recovery process from COVID-19, and the challenges faced by patients, is critical for the development of effective interventions and support programmes to help individuals manage their symptoms and improve their overall well-being. Comparisons with critically ill non-COVID-19 samples suggest that disability outcomes may be similar (Hodgson et al., 2022). Nonetheless, patients’ uncertainty about the virus and the effects of the quarantine measures implemented seem to have had consequences for patients’ perspectives and their potential for recovery (Sun et al., 2021). Patients recovering from COVID-19 report psychological distress related to the unknown nature of the virus (Engwall et al., 2022). They may also experience that family relationships are affected and may have difficulty resuming their daily lives (Wallin et al., 2022). A recent thematic analysis of COVID-19 survivors found that societal restrictions, fear and communication problems due to the pandemic added to the challenge of recovery, but that social and professional support and information were found to be facilitating factors (Bench et al., 2022). In summary, there is reason to believe that the recovery process after severe COVID-19 is influenced by the dynamics of the unfamiliarity and uncertainty surrounding the virus and may therefore be perceived differently from non-COVID recovery. As the COVID-19 pandemic continues, and with the risk that future pandemics may arise, exploring the experiences and perspectives of patients surviving the virus is essential to understanding the long-term consequences of recovering from a novel viral disease. This explorative study aims to examine patients’ experiences of receiving care on an ICU for COVID-19 and the subsequent rehabilitation process, with emphasis on facilitators and barriers to recovery.

## Material and method

### Design

This study is a part of a multicenter cohort study, which is a collaboration between two universities and two university hospitals in Sweden, aiming to explore patients’ psychosocial health after being treated for COVID-19 in an ICU (Halvorsen et al., 2023). Here, we report on an exploratory qualitative study within the prospective ICU cohort that included adult (≥18 years of age) patients admitted to intensive care for PCR-verified SARS-CoV-2 infection at two university hospitals in Sweden (Clinical Trials Registration: NCT04316884). All patients who survived intensive care at these two units were offered to take part in a follow-up at three to six months after discharge, and one year after discharge (Clinical Trials Registration: NCT04474249). At the first follow-up, patients were screened to take part in the qualitative study we report here, and then selected patients were included in the study. For this study, an explorative and inductive design was used, following the Consolidated Criteria for Reporting Qualitative Research Checklist (COREQ).

### Settings and participants

Participants were recruited from two university hospitals in Sweden. All patients admitted to the ICU due to COVID-19 from March 2020 to April 2021 and who enrolled in the ICU follow-up were invited to participate in the cohort study, either by a healthcare professional during the ICU follow-up or by mail. A total of 150 patients were included in the follow-up. They were informed that a selected sample of participants would be recruited for a research interview. A purposive sampling method was used, which aimed to include participants representing a range in sex, age and length of stay on the ICU. The inclusion criteria were patients of adult age (≥18 years), who had received care in an ICU due to COVID-19 between March 2020 and April 2021, and had the ability to understand and speak Swedish. Saturation was discussed during recruitment and data collection, and in total, 20 participants took part in an individual interview; however, 18 interviews were included in the analysis as two of the interviews had to be excluded from the analysis due to technical issues. A further five participants were contacted; however, these participants did not take part, e.g., due to stroke, poor memory, mental illness, or not responding to attempted calls.

### Ethical statement

The ethical principles for this research project followed the Helsinki Declaration guidelines (World Medical Association, 2018). The study has received ethical approval from the Swedish Ethical Review Authority, no. 2022–07064–02 and no. 2020–05758. The participants provided written informed consent prior to the interview after having received oral information about the study.

### Data collection

Data were collected using semi-structured interviews. The interview guide was pilot tested by the authors (RE and LH) with two patients who had received care in an ICU due to COVID-19. The interview guide was subsequently modified slightly before being used in this study; the guide is included as supplementary material (Supplement online material, 1). The interviews were performed 3–13 months (*M*: 9.3) after discharge from ICU, by one of two psychologists, one of whom is one of the authors (SN). The psychologists contacted the participants by phone to make an appointment for the interview. During the call, the participants received more information about the study, a written consent was sent to the address provided by the participants and the psychologists informed about their role in the project, and about the project in general. The relationship between the interviewer and the participants was strictly professional, and the psychologist had not met the patient before at the ICU. Due to the pandemic, all interviews were carried out via a reliable video platform or by phone, according to the participant’s preference. The participants were asked to reflect on their experiences of their stay in the ICU, their recovery during the subsequent rehabilitation phase with a focus on psychosocial aspects, and their perceptions of the support they received from public services, friends and family. Since the interviews were conducted digitally, we cannot fully rule out that anyone else was present during the interview. The interviews lasted between 20 and 95 minutes (*M* = 48 min). All interviews were audio recorded and transcribed verbatim by a professional transcribing service. Additional demographic data on the participants were collected from the survey in the cohort study.

### Data analysis

Thematic analysis was used to analyse the interviews in order to identify patterns in the data (Braun & Clarke, 2006). The transcripts were read several times by the first, second and third authors (RE, SN and LH) to gain a sense of the whole. The same three authors then coded two of the interviews individually and compared their codes to ensure similarity in the analysis. After this, each of the three authors coded a part of the remaining interviews individually. During this process, the three authors engaged in collaborative reflective discussions while maintaining a focus on the aims of the study, the research questions and the method used. The next step was to compare all the codes in the dataset and discuss similarities and differences between the codes before grouping them into preliminary themes derived from the data. When preliminary themes had been created, the other authors read a randomly chosen transcript to gain a sense of the data and provided their insights; all authors then discussed the analysis process, after which three authors (RE, SN and LO) finalized the analysis. No triangulation or feedback on the study findings was performed with the participants after the interviews. To enhance trustworthiness, the final analysis was discussed in the research group until all authors reached a consensus.

## Results

The age of the 18 participants varied from 32 to 76 years (*M* = 62), 14 were men, 13 were living in a relationship, and 9 were retired. The length of their stay in the ICU ranged between 4 and 51 days (*M* = 20; *Mdn* = 13; Table I).

**Table I.** Characteristics of the patients.

Characteristics	Patients, *n* = 18, n (%)
*Gender*	
Men	14 (78)
*Age*	
Min-Max; Mean	32–76; 62
*Occupation*	
Pensioners	9 (50)
Sick-leave	4 (22)
Working part-time	2 (11)
Working full-time	3 (17)
*Civil status*	
Married or committed non-marriage relationship	13 (72)
Single	4 (22)
Undisclosed	1 (6)
*Days in ICU*	
Min-Max; Mean; Median	4–51; 20; 13
*Sedation*	
Yes	14 (78)
*Invasive ventilation*	
Yes	11 (61)
*Days of invasive ventilation*	
Min-Max; Mean	1–41; 13
*Non-invasive ventilation*	
Yes	18 (100)
*Pandemic waves*	
Wave 1 (spring 2020 – summer 2020)	7 (39)
Wave 2 (fall 2020 – winter 2021)	0 (0)
Wave 3 (spring 2021 – summer 2021)	11 (61)

The thematic analysis resulted in two themes: *“An isolated world with silver linings”* and *“Recovery in the wake of the pandemic”* with two and four subthemes, respectively (Table II).

**Table II.** Themes and subthemes.

An isolated world with silver linings	
	Good care despite the circumstances
	Living between dreams and reality
Recovery in the wake of the pandemic	
	The public services did their best to help
	Taking one step at a time
	Adapting to a new self
	Appreciation, love and support from family and friends

### An isolated world with silver linings

#### Good care despite the circumstances

The participants reported that the care they received in the ICU was good and it had given them a positive impression of Swedish health care. The participants also expressed gratitude towards the healthcare staff and the way they handled the care situation despite the ongoing pandemic. Some participants said that they felt safe and secure and were never afraid during their time in the ICU, and that they had been well taken care of by the staff:

And you felt so incredibly safe with the staff. And they were so nice. They did everything. When I was a bit better, after a week or so, the doctor said “Yes, but she needs to be able to see outside.” And then they rearranged pretty much the entire room to be able to turn the bed so that I could look out of the window (woman, 66 years old).

The healthcare staff encouraged the patients to start physical training with a physiotherapist, and to receive support from a social worker regarding psychological issues. Participants reported that their time in the ICU had been relatively good despite the circumstances, but that the longer it went on and the healthier they became, the more tedious it was perceived to be. Since visitors were prohibited from coming to the ICU due to the pandemic, the staff provided patients with their only company and this could create feelings of isolation and loneliness:

I was never alone in the ICU, but I felt alone because my family wasn’t there. Even though I always had the staff around me … Yes, it’s a different kind of loneliness you feel than if you had been completely alone in a room (woman, 32 years old).

A few participants had difficulty remembering what had happened during their stay in the ICU because they had been sedated:

And it’s just gone. I have no … I have read about other people’s experiences there and I really don’t have any recollection of that period (man, 61 years old).

#### Living between dreams and reality

The participants described experiencing many physically demanding symptoms from COVID-19 during their time in the ICU. Having another underlying illness could also make their stay in the ICU more problematic.

*But it was not a good combo, asthma and covid, it really wasn’t* (man, 70 years old).

The participants described fluctuations in pulse rate, oxygen saturation that decreased with activity, loss of muscle strength, and their body feeling like “jelly”. Many participants also reported experiencing pain in different parts of their bodies, especially when breathing. The non-invasive ventilator was described as feeling like a “storm” in their head and, since the oxygen mask was attached to their face for long periods of time, some patients experienced it as claustrophobic. This was described by one patient:

I was given a kind of oxygen there that was like over my whole face, you could say. It felt as if you were suffocating (woman, 64 years old).

Some participants described their time in the ICU as a personal crisis. This started with the realization of how sick they were, but also with them feeling worried or sad because of the situation, as one participant said:

Gradually, you started to understand. That was the toughest thing mentally, when you started to realise how bad it was (man, 68 years old).

A few participants said they had felt that they would be better off dead, with suicidal thoughts coming and going:

Then I was feeling so bad that I remember an hour … a few hours … I don’t know how many hours, but I felt so bad that I asked to be allowed to die. Because I couldn’t take it anymore (man, 32 years old).

One participant also found it especially difficult to think about others who had been cared for and that people with COVID-19 may have previously died in the room they occupied.

Many participants said that they felt as if they were in another dimension, between dreams and reality, which resulted in a strange perception of time. Some also described experiencing hallucinations or very strange dreams that were unreal and scary. One participant described this as follows:

Then consciousness kicked in, but not reality, because I had very, very intense dreams. I still remember some of them to this day. And then eventually reality set in, bit by bit. So, you lived in some sort of phase between dreams and reality (man, 68 years old).

Participants reported being worried about their relatives at home who had no knowledge of what was happening to them in the ICU. Some participants described feeling that it must have been worse for their family members who were waiting for them to recover and be discharged from hospital, for example:

But it must have been very hard for them. I can imagine that it was maybe almost worse for them. I was in the middle of it and knew what was happening. Especially … I know that my daughter told me that when I went into ICU she almost broke down. And my husband was also extremely worried. But they had daily contact with the doctors and so, but … But then, as I got a bit better, things improved, because then we could talk on the phone (woman, 66 years old).

However, participants reported how their health improved gradually from day to day and that the feeling of not succumbing to the illness was perceived as a good sign.

### Recovery in the wake of the pandemic

#### The public services did their best to help

After receiving care in the ICU some participants described how they were then transferred to many different wards in the hospital before discharge. This was troublesome due to the ongoing pandemic and also because they had to meet new healthcare staff.

*And then I was moved between so many different wards, so I actually didn’t know where I was, which ward I was on* (woman, 71 years old).

The participants described how, after discharge, they received help from the hospital in many different ways, and from various public services and healthcare professions. Some participants were enrolled in a post-covid outpatient clinic, whereas others had to register for this themselves.

Many participants received help from physiotherapists at a health care centre or from staff in the municipality. The physiotherapists made home visits to many participants and provided individualized support, which the participants said they appreciated.

*I’ve had a really good physiotherapist. At the beginning she came two to three times a week. And she also stopped me overdoing it. She was very good* (man, 32 years old).

Some participants said that they came into contact with a psychologist or a social worker regarding their recurring nightmares, the guilt of being ill, or for help with accepting their situation. A few participants had been given the contact details of the psychologist or social worker but had declined to make contact. Many participants also received support from physicians at health care centres. Some described this as positive and helpful, while others reported that there was a lack of continuity.

I got good help from the doctor up until last spring. Since then it’s been a bit up and down. I have a new GP. Now they’ve started taking some tests again and we discuss medication and everything. The first one was so busy he never had time. Now I’ve got another one who I’m going to see in a week or so, so I hope he’ll be better. (man, 32 years old)

Other participants received visits from home care staff in their municipality who helped with laundry and cleaning during the first weeks after discharge.

For those still in employment, getting back to work after being in the hospital was described as a slow process, and some had been on sick leave for almost a year. Some participants described the negative effects of returning to work early in the rehabilitation process. In addition, some experienced a lack of understanding from their employer and the Swedish Social Insurance Agency:

I got a bit angry about the way they acted. You’ve just come home from the hospital and may have been close to death, I don’t know. Then they start talking about going to work, it’s so horribly stupid that it’s crazy (man, 55 years old).

Being self-employed was also described as challenging; however, it was also described as facilitating being able to go back to work at one’s own pace, since being at work was motivating and decreased the opportunities for ruminative thoughts.

Many participants had wanted more support from the public services and described how this had been inadequate and sporadic:

I would have needed … some more help when I was discharged from the hospital. And maybe already when I left ICU. That you could get to talk to someone there who could give more answers. I was at a hospital visit and saw an infectious disease doctor last autumn and went through everything. And then I asked him a few things. But he didn’t really know. But he could anyway give me some answers. But I would have needed that earlier, I think (woman, 66 years old).

At the same time, participants said that they understood that a pandemic is an unusual situation and that the public services had done everything possible under the circumstances. Not one participant expressed bitterness or anger, but rather patience regarding the whole situation:

I was lucky as I came in one year after it had started. And then people knew much more about how to treat people who had covid. Yes. It was actually a staff member who said, “well, you were lucky to come now. Because now we know so much more about how to do things than in 2020” (woman, 64 years old).

However, a few participants said that they felt they had been forgotten and wished that they had developed another illness instead of COVID-19 so that they could be given better outpatient care:

But from having been so well taken care of until it ended, or so it felt, last summer. I thought “have I been declared healthy now or have I been forgotten?” (man, 68 years old).

#### Taking one step at a time

The participants described how they were not fully recovered from COVID-19 when they returned home and that during the first weeks at home they felt shattered. Almost every participant described how their physical condition was impaired. They talked about increased shortness of breath, being barely able to walk, not having the energy to perform activities, and having to rest for several hours after doing simple household chores. One participant described taking the steps in the stairwell to his home as being very challenging:

It took me almost two weeks to be able to leave the apartment, because there are steps, there’s no elevator. There are two stairs down. And going down the stairs … you get a bit dizzy, but going up the stairs, they were hellish to go up (man, 58 years old).

During the interviews, many participants reported needing to take a nap during the day or having sleeping problems and fatigue. One participant said:

So, I’ve found it really difficult to sleep at night and I wake up several times during the night and check whether I’m alive or that my body still works (man, 62 years old).

Participants also described very severe muscle soreness. Low oxygen saturation levels were still a problem after discharge, as were impaired balance, dizziness and the loss of sensation in various parts of the body. Other symptoms included difficulty urinating and therefore needing a catheter, tinnitus, changes in taste perception, reduced sense of smell, weight loss, and difficulty speaking due to hoarseness. Some participants expressed concerns about whether their symptoms were a result of COVID-19 or if they were experiencing new symptoms due to ageing:

Before you could do whatever the hell you wanted, but that… But that also changes with age, that has an effect. Maybe it’s not just covid (man, 70 years old).

In addition, they described the process of regaining power and strength in their body as being very slow, which surprised some of the participants. As time passed, participants regained their energy and described life slowly returning to normal each day, little by little.

However, some participants stated that the symptoms fluctuated in presence and severity, which made it difficult to predict how the next day was going to be since life seemed to be constantly changing:

I notice that … I never know when I go to bed at night how I’ll feel the next morning? It’s often worst in the mornings. Getting out of bed can be hellish. And then I’m like a zombie all morning some days. And then it can ease up a bit. It varies so much, up and down from day to day (man, 68 years old).

The participants perceived that factors that facilitated rehabilitation were having a good level of fitness before their illness and being able to make use of their previous strength, exercising regularly during rehabilitation even if it could be difficult, and thinking positively about exercise despite the pain. Many participants set personal goals for themselves, such as walking to the mailbox every day and increasing their walking distance gradually. One participant described this as follows:

But I tried to take a walk every day. And I walked a bit further each day. So after a few … I walked between every park bench. It was maybe 200 metres and I sat down on a park bench, rested for ten minutes, then I went to the next park bench, sat there and rested for ten minutes and so on (man, 32 years old).

Other participants described how the rehabilitation process was facilitated by having to exercise their pets, being at their summer cottage, working, gardening and doing everyday household chores.

Almost all the participants said that, at the time of the interview, they were almost fully recovered, doing the same things as before COVID-19, such as taking the stairs with no problems, easily taking the dog for a walk, and performing household chores again.

#### Adapting to a new self

The majority of the participants reported having problems with their mental health and with existential thoughts during the recovery process. Participants said that being affected by COVID-19 and needing care on the ICU had made them aware of being mortal, and how life cannot be taken for granted. This had led to participants having many existential thoughts that they had never experienced before. After being discharged from the hospital they were left feeling they had been granted more time and had gained a newfound appreciation for life.

But you have some thoughts about life, existential aspects and everything afterwards somehow. I think so anyway. … I had a near-death experience when I was at my worst in the ICU, which I’ve anyway thought about a lot. I probably do that more now. What happens afterwards, and what about the soul, and all those kinds of things. That’s what I think about (woman, 66 years old).

The recovery period was described as a time of isolation, partly because of the pandemic restrictions but also because of their lack of energy. Some participants described how they became less sociable and postponed meetings, gatherings, and other things that were important to them. As one participant said:

But that it’s given me a sense of vulnerability, I know I can’t manage social things in the same way as I did before, which has led to me living a less sociable life nowadays, I see my friends and my family less than I did before. Which, when I think about it, makes me sad (woman, 32 years old).

A few participants also reported that the reason for their isolation was a fear of becoming ill again, so they avoided meeting others, even close family.

The tiredness that the participants experienced was not just physical but also mental, described by the participants as “brain fog”. During the interviews they said that they had difficulty finding words, were sensitive to sound and light, were less resistant to stress, had difficulties with memory and concentration, and felt irritable more often. One participant described the following:

Yes, I think my brain is tired, actually. Because I’m so sensitive to noise, when people are talking a lot. Now people have come back to work, before we worked remotely. And I notice it, everyone’s talking, it’s becoming a bit too much. I’m going to buy a pair of headphones that actually block out noise, I think. And that’s really the way I feel. I’m less stress resistant too, actually (woman, 64 years old).

The participants also described an increased mental sensibility, which was expressed through crying more often, and feeling increased worry about the future, their symptoms, and the people they care about. Some said that they felt sad or depressed, that things that gave them joy in life before COVID-19 no longer did so:

But it’s because I feel a bit lethargic. I don’t feel any real kind of joy and I don’t feel sad either, but… yes. It is a bit harder to get started on doing things (man, 62 years old).

For a few participants, these emotions led to thoughts of them not wanting to live anymore.

In contrast, some participants expressed resilience in their way of thinking, telling themselves to accept the situation and regain their lives; but also to accept that they may not recover fully, to learn to listen to their body, to stress less and hope for better times. The participants stated that factors that facilitated the mental rehabilitation process were finding motivation and having meaningful things to do in everyday life, being able to see things positively, and trying to get back to how life was before. One participant summarized this as follows:

I’m happy I survived. And I’ve tried to make myself become as well as I can possibly be. It’s my goal to, yes, eat well, exercise and do everything in my power to be as good as and better than I was before. That’s what the goal is to be able to … ski again, which I love doing. And that… I’ve been skiing now this spring, just carefully. But to be able to get back to how it was before when I had the energy. So that’s the goal. Absolutely (man, 32 years old).

Some participants tried to find meaning in, or positive outcomes of, becoming so ill with COVID-19, as one person described it:

Several people said [to me], “I would never have had the vaccine if you hadn’t been sick”. Yes, so it was of some use anyway (man, 64 years old).

#### Appreciation, love and support from family and friends

Participants described how, when they were discharged from hospital, people around them were happy to see them again which led to many positive reactions and feelings of appreciation. Friends called to see how they were doing, sent cards and flowers, and family and neighbours brought groceries. In the first months the participants received lots of support and felt that they were given the care and love they needed from family and friends. One person described the situation as follows:

I received an incredible amount… Some came home just to be there… Because of the way it was there was no direct physical contact, because that wasn’t such a good thing in the context of covid. But still, there were many who … by phoning and visiting and leaving flowers. So, I had so many flowers at home. So that phone calls and visits from… My siblings were here a bit more often maybe. We were sitting out on my balcony when I… it was my birthday just as I got home, then they came. So, they were… They weren’t all here at the same time, but they were… They had to take it in turns to come, so to speak. But still, it was quite a lot. I met a lot of people anyway that way (man, 76 years old).

However, some participants reported being almost dependent on their family members in the first weeks in order to be able to take care of themselves, e.g., needing help with showering and household chores, which could provoke feelings of guilt. One participant reasoned:

Imagine if I’d lived alone and had no one and had been so sick and came home, it wouldn’t have worked. So that then… when I came home, I needed someone with me 24/7. Someone… I would… I couldn’t even cook and didn’t… I couldn’t manage to take a shower by myself, I didn’t do anything. So, without them [his parents] I wouldn’t have managed. My partner showered me, cooked my food, and my parents did too. And fixed everything. It’s so stupid. But my days… I couldn’t do anything at the beginning. So, I was more like a child. It … They had to take care of everything. And without them I would have… Either I’d have had to stay in hospital or, yes, I don’t know what would have happened. So that yes, no, they took care of my everyday life at the beginning (man, 32 years old).

Participants with grown-up children reported that their children were a very important source of care, support and love during the recovery process. Many participants felt closer to their families after the experience, which led to them becoming better at communicating, not taking them for granted, and also placing greater value on the time they spend together. One participant said:

Although now, since I came home, we notice that we take care of each other much more. That’s the good thing about it all, so to speak. So, we have become even closer I can say, both my girls and their families and us here. You realise how quickly life can change (man, 76 years old).

However, the pandemic prevented social interactions to a great extent and some participants described how the care and support they received initially decreased over time. Feeling tired, being unable to help with household chores, and being sensitive to light and sound could also create tension and conflicts within the family.

The participants reported that factors that facilitated their rehabilitation were having family and friends, hanging out with others, and being able to laugh and joke. However, talking about what had happened, and the experience of this time and process for everyone involved, were also facilitating factors. Some participants described how putting their experience into words helped them to understand what they had been through. One way of talking about and understanding their time in the ICU was through reading the diary that had been written there. This contained a lot of information that was valuable for both the participants and their relatives. One participant described how they use it:

But I also took the opportunity to read through the diary I received from the ICU. I couldn’t bear to read it all at once, because I got very upset when I read it. But my partner and I read it a few days at a time, and then I also read my medical records. My family kept a diary when I was sedated, so did my partner. So, I have looked at all those and tried to process what I’ve been through. And I’ve been… So, it’s like this, I’ve been very moved when I’ve read the things that they’ve written or when I’ve gone through the messages that they’ve sent and so forth. But it’s been a very nice feeling throughout, because it’s become obvious that I have people around me who care a lot about me. That’s been lovely. (Woman, 32 years old)

## Discussion

The findings from this explorative study indicate that patients who were cared for in an ICU during the COVID-19 pandemic felt safe but also experienced a sense of crisis and vulnerability, highlighting the fragility of life. After being discharged from the ICU, the participants expressed gratitude for the efforts made by the public services to assist them during their rehabilitation process, even if they were unsure about how to do this. Despite the support received from various institutions and healthcare professionals, physical rehabilitation was a slow process with frustrating day-to-day fluctuations. Participants described feeling isolated, fatigued and emotionally sensitive. They were also adjusting to a new self, with limited energy and lingering symptoms as a result of COVID-19 and possibly due to ageing. Receiving love and support from family and friends was crucial for the recovery process, as was the diary written by the ICU staff, which proved valuable for their mental recovery.

Even in normal circumstances, a stay on ICU is an extremely stressful and generally unpleasant experience for critically ill patients. The visiting restrictions in hospitals during the pandemic prevented the customary regime that involves the presence and participation of relatives. This is an important part of the care of patients in the ICU in order to lower the patient’s risk of anxiety and psychological crises, and strengthen them in the rehabilitation process (Alsharari, 2019; Jeffs et al., 2017). The results of this study also highlight an important new dimension concerning this aspect, namely that the patients worried not only about themselves but also about their relatives.

The recommendations for follow-up after discharge from the ICU is that the patient and their relatives are offered a meeting at the ICU follow-up clinic. This gives patients an opportunity to understand their physical and mental impairments, and receive an explanation for the hallucinations and nightmares that often occur during ICU care, which is an important element of further processing and coping with the effects of their ICU stay (Hanifa et al., 2018). During the pandemic, most of the ICU follow-up clinics in Sweden were suspended because healthcare personnel were needed to provide care for patients on the ICU. Most of the surviving patients, therefore, did not receive a follow-up meeting after discharge from the ICU. Some of the patients in the current study expressed the need for explanations and made use of different coping strategies in the recovery process. The participants received many different kinds of care, from various places and healthcare professionals, and expressed this as a burden for the rehabilitation process. This is in line with previous research on COVID-19 and the rehabilitation process after ICU care (Bench et al., 2022). However, findings from the present study show that participants did not feel bitter but were instead understanding of the system having been overwhelmed.

The experiences of the physical aspects of the rehabilitation process in this study were described as a need to take “one step at a time”. This is consistent with other research on rehabilitation after COVID-19, such as Engwall et al., 2022, describing the recovery as “two steps forward, one step back” since symptoms emerged and fluctuated from day to day. All of these symptoms and the slow process of recovery affected everyday life for the participants in the present study. Findings from this study and other COVID-19 studies (Bench et al., 2022; Huang et al., 2021; Lopez-Leon et al., 2021; Parker et al., 2021) show that managing fatigue and physical weakness is a large part of the rehabilitation process after a SARS-COV-2 infection. However, during this slow process towards better physical health, participants described many activities and coping strategies that they used in order to progress in their rehabilitation, such as physical activity, thinking positively, setting goals to work towards, having a pet to take care of, and staying in a second home (e.g., a summer cottage, which is common in Sweden).

Strategies and standardized care procedures for those who were admitted to the ICU due to COVID-19 were lacking during the initial stages of the pandemic. Previous research indicates that, as a result, these individuals have developed expertise in their own physical and psychological experiences and what they require for a good healthy recovery (Engwall et al., 2022). Considering the various symptoms they may experience, the rehabilitation process for ICU patients surviving COVID-19 is complex and requires a holistic approach (Humphreys et al., 2021). These patients have learned to understand their needs differently compared to those receiving regular outpatient care, and this understanding should be taken into account in their recovery.

Previous research shows that being affected by COVID-19 and admitted to an ICU increases the risk of mental health problems such as anxiety and depression (Huang et al., 2021; Morin et al., 2021; Parker et al., 2021). Even if the present study did not explicitly explore the psychological impact of being admitted to the ICU due to COVID-19, the participants described an increased emotional sensibility, expressed through crying more often, having fears about the future, and feeling lethargic or depressed. In the present study, and also another study about recovery after COVID-19 (Bench et al., 2022), participants were offered the possibility of psychological and psychosocial support from health care centres in their municipality, but only a few participants accepted. The nature of the patients’ difficulties in a prolonged recovery process suggest that psychosocial services as part of community care, or telehealth services, may increase uptake of these psychosocial services to the benefit of the patients, who may feel that visiting healthcare facilities is too burdensome. Social interactions with family and friends seemed to be of the utmost importance during rehabilitation after a severe COVID-19 infection (Engwall et al., 2022). In contrast, previous research has shown that this long recovery process could be a burden from a social perspective for both the patient and their family (Bench et al., 2022). The findings from the present study suggest that the rehabilitation process would clearly be more difficult without the support of family and friends.

This study has limitations that are important to consider when interpreting the results. The participants comprised a heterogeneous group, although were mainly men retired from the workforce. They had been admitted to the ICU at different time points during the pandemic, introducing potential differences with respect to the ICU care they received and the subsequent rehabilitation processes. In addition, the participants were interviewed at different time points after discharge. Even though the participants in this study had experienced a potentially traumatic event, most reported that they had regained high levels of function and had mainly low levels of affective problems, suggesting a high level of resilience in the sample. The findings of this study are, therefore, unlikely to reflect the experiences of all patients admitted to an ICU in Sweden due to COVID-19. The steps of the research process have, however, been described as transparently as possible, contributing to dependability. Another strength of this study is the solid thematic analysis, where the verbatim quotations from the participants added to the study’s credibility. This study contributes to a deeper understanding of the studied phenomena from the patient’s perspective, while being consistent with previous research on recovery and rehabilitation after being admitted to an ICU due to COVID-19.

In conclusion, this study increases our knowledge about the experiences of patients with COVID-19 who received care in an ICU, and of their recovery process, in the wake of the pandemic. Positive and negative aspects of their experiences during their stay on the ICU were identified, where participants expressed gratitude for the care they received while also experiencing feelings of isolation and loneliness. This study also highlights the various ways participants received help from different healthcare professions and public services, as well as their perceptions of the support received during recovery after discharge from the ICU. Some expressed a wish for more support from public services, indicating a potential area for improvement. Continued follow-up could be valuable in helping to identify any ongoing physical, mental or cognitive health needs of these patients. The study findings may also contribute to improvement of the care and support provided to patients with COVID-19, or other virus infections in future pandemics, during their hospital stay and after discharge. Overall, the study presents information about the challenges of recovering from COVID-19, emphasizing the importance of continued support from health care and the public services. It provides valuable insights into patients’ experiences of COVID-19 and can inform future healthcare strategies and policies to prepare for future pandemics.

## Supplementary Material

COREQ_Checklist_231228.pdf

Supplemental online material.docx
