# Supplementary material for: Surviving COVID-19: patients’ experiences of care and path to recovery
Source: Int J Qual Stud Health Well-being. 2024 Jan 7;19(1):2301953. doi: 10.1080/17482631.2024.2301953 (PMC10773674; doi:10.1080/17482631.2024.2301953)
Supplement: Supplemental online material.docx [file ZQHW_A_2301953_SM9174.docx]

# Supplemental online material

**Open interview questions:**

1. Can you summarise your time on the ICU and how you experienced it?

2. Tell me about the period from when you were discharged from the ICU until today.

3. How would you describe your recovery since your time on the ICU?

4. Is there anything that has made things easier for you during your rehabilitation?

5. Is there anything that has made things difficult for you during your rehabilitation?

6. Can you describe how you feel today?

7. Can you describe what a typical day is like for you?

8. What are the 2-3 biggest differences between how you feel today and how you felt before you got Covid-19?

9. If you regard yourself as having recovered, approximately how long did it take before you felt fully recovered?

10. What support do you consider you have received from public services? (formal support)

11. What support do you consider you have received from family and friends? (informal support)

12. Do you think that what you’ve been through has given you any positive experiences?

13. Is there anything else that you want to tell me that I haven’t asked about during this interview?
